# Prélèvement de plaquettes pour la chirurgie cardiaque: première expérience de d’aphérèse à l’Hôpital Général de Douala

**DOI:** 10.11604/pamj.2018.31.41.16571

**Published:** 2018-09-20

**Authors:** William Ngatchou, Isabelle Dreezen, Felicité Kamdem, Gisèle Imandy, Cecile Okalla, Albert Nkana, Jean Pierre Hacquebard, Pierre Origer, Joseph Sango, Daniel Lemogoum, Sidiki Mouliom, Anastase Dzudie, Henri Ngote, Romuald Hentchoya, Junette Metogo, Olivier Germay, Eugène Belley Priso, Jean Luc Jansens, Henry Luma, Tome Najdovski

**Affiliations:** 1 Département de Chirurgie, Faculté de Médecine et de Science Pharmaceutique de Douala, Cameroun; 2 Croix Rouge belge, Belgique; 3 Service de Médecine et Spécialité Faculté de Médecine et Science Pharmaceutique de Douala, Cameroun; 4 Service de Médecine Hôpital Général de Douala, Cameroun; 5 Laboratoire Hôpital Général de Douala, Cameroun; 6 Service d’Anesthésie CHU St Pierre de Bruxelles, Université Libre de Bruxelles, Belgique; 7 Service d’Anesthésie Réanimation Hôpital Général de Douala, Cameroun; 8 Direction de Hôpital Général de Douala, Cameroun; 9 Hôpital Erasme de Bruxelles, Université Libre de Bruxelles, Belgique

**Keywords:** Chirurgie cardiaque, aphérèse, plaquettes, Trima, transfusion, Douala, Key words, Cardiac surgery, apheresis, platelets, Trima, transfusion, Douala

## Abstract

La chirurgie cardiaque sous circulation extracorporelle (CEC) est régulièrement associée à la perte d’une quantité importante de sang. Une bonne anticipation de ces pertes sanguines et une bonne hémostase peropératoire sont reconnues comme moyens permettant de limiter ces saignements post opératoires. Jusqu’à ce jour, la compensation des besoins en plaquettes des patients opérés sous CEC dans notre service se faisait par transfusion de sang total ou de concentrés plaquettaires prélevés à partir de plusieurs donneurs. Nous rapportons la première expérience de prélèvement de concentrés plaquettaires par la technique d’aphérèse à l’Hôpital Général de Douala.

## Abstract (en)

Cardiac surgery with extracorporeal circulation (ECC) is usually associated with the loss of a significant amount of blood. Adequate prophylaxis against blood loss and good perioperative hemostasis are known as processes limiting postoperative bleeding. Until now, the need for platelets in patients operated with extracorporeal circulation in our Department has been compensated for by total blood transfusion or platelet concentrates collected from several donors. We here report our first experience with platelet concentrate collection by apheresis at the General Hospital in Douala.

## Introduction

La chirurgie cardiaque sous circulation extracorporelle est régulièrement associée à la perte d’une quantité importante de sang. Une bonne anticipation de ces pertes sanguines et une bonne hémostase peropératoire sont reconnues comme moyens permettant de limiter ces saignements post opératoires [1]. La transfusion des composants du sang débutée dans les années 1960 grâce à l’utilisation de poches multiples en plastique a permis de développer les techniques chirurgicales, en particulier la chirurgie cardiaque dans les décennies 1970-80 et la chimiothérapie des maladies malignes au cours des décennies 1980-90 [2]. La compensation des besoins en plaquettes des patients peut se faire soit par la transfusion de sang total, soit de concentrés plaquettaires prélevés à partir de plusieurs donneurs, soit d’un seul donneur dans le cas de l’aphérèse [1, 2]. A cause des contraintes financières, de l’inexpérience, de la non maitrise de la chaine de froid, de l’absence de capacité de stockage, la majorité des pays d’Afrique subsaharienne utilise le sang total comme unique produit sanguin [3-6]. Ce constat est alarmant car bien que le risque de contracter, à la suite d’une transfusion d’un concentré de globule rouge ou de plaquettes, une infection due à un micro-organisme soit devenu exceptionnel dans les pays développés [7, 8], il reste un problème de santé publique en Afrique Subsaharienne (ASS) [3-6]. Pour faire face à cette situation certains pays d’ASS comme la Namibie ont développé des techniques alternatives permettant la limitation du nombre de donneurs [9]. La technique « buffy coat » précédemment utilisée pour l’obtention des plaquettes à partir de plusieurs donneurs a été abandonnée pour l’aphérèse qui ne nécessite qu’un donneur unique [9, 10]. Le succès de l’implantation de cette technique dans les pays comme la Namibie et le Rwanda, l’augmentation de la demande en composés sanguins grâce au développement de la chirurgie cardiaque et de l’onco-hématologie dans notre institution ont décidé les autorités de l’hôpital à saisir l’opportunité qu’offrait le service du sang de la Croix Rouge de Belgique pour l’acquisition de cette technologie.

## Patient et observation

**Figure 1:**
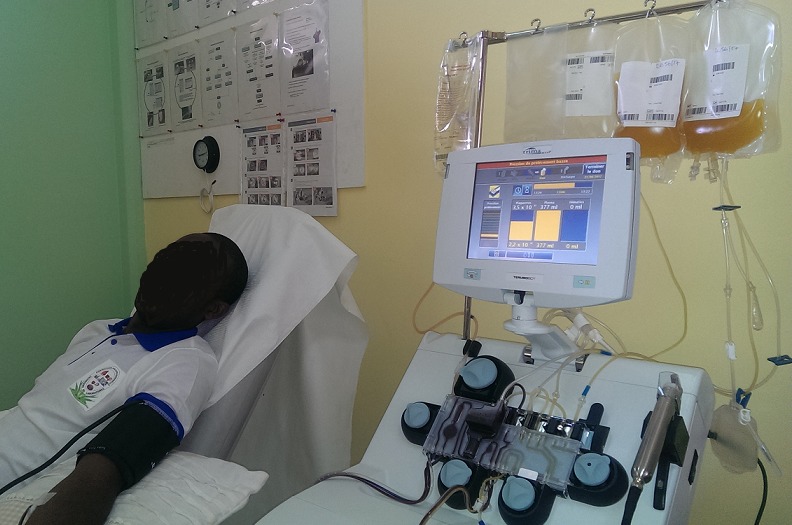
Patient en pleine procédure de don

Il s’agit d’un donneur volontaire non rémunéré de 28 ans (poids 75 Kg, taille 174 cm) sans antécédent particulier. Il était à son premier don de sang. Son groupe sanguin est du groupe A positif. Sa biologie (numération formule sanguine) montrait un taux de globules blancs à 3.2 10^3^/μL (valeurs usuelles = 4-10 10^3^/μL); globules rouges à 5.58 10^6^/μL (valeurs usuelles = 4-6 10^6^/μL); plaquettes 233 10^3^/μL (valeurs usuelles = 150-400 10^3^/μL); hémoglobine 14.7g/dl (valeurs usuelles = 12-17g/dl); hématocrite 42.1% (valeurs usuelles = 34-57%). Ses sérologies VIH, Hépatite B, C, la recherche d’hémoparasites étaient négatives. Les tests d’hémostase, la fonction rénale, les tests hépatiques et la calcémie étaient normaux. Le prélèvement s’est fait la veille de l’intervention (Figure 1). Le donneur a suivi la filière habituelle du don de sang recommandée par le ministère de la Santé (consultation, analyse sanguine complète, information éclairée) et le prélèvement s’est fait à l’Hôpital Général de Douala qui est un des centres agréés au Cameroun.

En ce qui concerne la procédure, elle a commencé par un questionnaire, ensuite, les étapes de la procédure ont été réexpliquées au donneur et le consentement signé. Il a été installé dans un fauteuil. Après désinfection, une aiguille a été introduite dans une veine de bon calibre de l’avant-bras du donneur. Cette aiguille est pré-connectée (afin de disposer d’un système hermétique) à un kit placé sur le séparateur automatisé Trima Accel^TM^(Terumo BCT Europe). Ce dernier est un système automatisé de collecte de sang conçu pour la collecte de composants sanguins à des fins de transfusion. Il peut collecter des combinaisons de produits sanguins: concentrés érythrocytaires (déleucocytés ou non), concentrés plaquettaires et plasma provenant d’un don unique. Ce système permet d’optimiser le nombre et le type de composants collectés à partir d’un nombre limité de donneurs tout en générant des produits sanguins de qualité destinés aux patients.

Le système Trima Accel^®^ utilise une centrifugeuse à flux continu pour séparer les principaux composants du sang. Le sang est prélevé auprès du donneur et mélangé à de l´anticoagulant (acide citrate dextrose). Ce mélange de sang et d´anticoagulant est pompé dans un anneau (placé dans la centrifugeuse), puis centrifugé à grande vitesse (3000 tours par minute pour les plaquettes) afin de séparer les composants sanguins. Deux forces entrent en jeu, la force centrifuge et la force de gravité afin de séparer les composants. Au niveau de la chambre de collecte, les lignes hématies, plaquettes et plasma extraient les composants séparés. Une ligne de contrôle régule l´interface entre les trois composants. Les plaquettes sont acheminées vers la chambre LRS (LeucoReduction System) où les leucocytes sont filtrés. Une fois déleucocytées, elles sont envoyées en dehors de la centrifugeuse. Les plaquettes sont ensuite acheminées dans des poches de produit. De la même façon, le plasma et les hématies sont récoltées dans une poche produit ou retournés au donneur. Un panneau avant sur lequel est déposée une cassette permet de contrôler et diriger le débit de sang dans le système. Ce panneau est composé de 5 pompes: la pompe à anticoagulant, celle de retour, de plaquettes, de plasma et de prélèvement ainsi que de deux détecteurs de niveaux, un détecteur d’hématies, de valves et de capteurs de pressions (centrifugeuse et prélèvement/retour). Celle-ci est composée d’un plateau comportant une rainure pour y déposer l’anneau séparateur. Cet anneau contient une chambre de collecte, une chambre LRS et des lignes d’arrivées, d’hématies, de plasma, de plaquettes et de contrôle.

Au total, un Concentré Unitaire de Plaquettes (CUP) (219ml équivalent à 7 unités soit 3.5 x 0.5 x 10^11^plaquettes) et une poche de plasma (347ml) ont été collectés chez ce donneur. La conservation s’est faite sous agitation constante dans une enceinte thermique calibrée entre 20 et 24°C. Etant donné que nous prélevons en 100% plasma, il n’y a pas eu de manipulation du produit prélevé si ce n’est un échantillonnage pour le comptage des plaquettes et analyses biologiques (bactériologie, comptage du nombre de GB). Le receveur âgé de 47 ans de groupe sanguin A Rhésus positif souffrait d’une insuffisance mitro-aortique sévère d’origine rhumatismale associée à une dilatation de l’aorte ascendante mesurée à 52mm. Sa biologie préopératoire montrait un taux de globules blancs à 6.5 10^3^/μL, de globules rouges à 4.1 10^6^/μL, de plaquettes à 275 10^3^/μL, d’hémoglobine à 11.8 g/dl d’hématocrite à 38%. Les sérologies VIH, Hépatite B et C étaient négatives. Le PTT était à 71% et le TC à 32 sec pour un témoin à 34 secondes. Le test de compatibilité pré-transfusionnel était normal. L’intervention a consisté en un remplacement de la valve aortique, de l’aorte ascendante proximale et d’une réimplantation des artères coronaires (intervention de Bentall) associé à une annuloplastie mitrale sous circulation extracorporelle au sang en normothermie. L’administration du culot plaquettaire et du plasma a eu lieu en fin d’intervention après contrôle de l’hémostase, arrêt de la CEC et antagonisation de l’héparine. Les suites opératoires ont été simples avec absence de transfusion supplémentaire en postopératoire et un taux de plaquettes à 291 10^3^/μL à la sortie.

## Discussion

L’intérêt de ce cas est qu’il est le premier à rapporter l’utilisation de la technique d’aphérèse pour le prélèvement de plaquettes en Afrique Centrale. En effet, alors que cette technique est répandue dans les pays industrialisés depuis les années 1970, seuls très peu de pays d’ASS l’ont introduite dans leur politique transfusionnelle [9, 10]. Cette partie de l’Afrique doit faire face à une haute prévalence de VIH et d’autres micro-organismes transmissibles par la transfusion [3-6]. Parallèlement, l’augmentation de l’incidence des cancers, des traumatisés graves liés à la violence routière et de nombreux conflits ainsi que le développement des techniques chirurgicales lourdes sur le continent comme la chirurgie cardiaque ont multiplié les besoins [11-14]. L’aphérèse à elle seule permet de résoudre ces deux premières difficultés car en n’utilisant qu’un seul donneur, elle limite le risque de transmission d’infection avec en plus une capacité de production par patient plus importante [8-10]. A l’hôpital Général de Douala, la multiplication des traumatisés graves due au phénomène des motos taxis [15, 16], le développement de la chirurgie cardiaque [17] et l’hémato-oncologie ont augmenté la demande en produits sanguins séparés [18]. Le système d’aphérèse utilisé dans le service de transfusion a été obtenu gracieusement du Service du Sang de la Croix Rouge de Belgique avec le soutien de la firme Terumo BCT. Ce partenariat qui inclut la formation des équipes locales a déjà été testé dans d’autres circonstances et est à encourager pour palier au déficit de techniciens qualifiés [5, 19]. A première vue, l’investissement pourrait représenter un frein pour l’acquisition de cette technologie mais l’expérience namibienne a montré au contraire que ces aspects pouvaient être maitrisés sur le long terme [**9**].

## Conclusion

Ce cas clinique montre que la production de concentrés plaquettaires par aphérèse est faisable dans notre contexte avec des avantages concrets en termes de capacité de production et de diminution du risque de transmission des micro-organismes.

## Conflits d’intérêts

Les auteurs ne déclarent aucun conflit d'intérêts.

## Contributions des auteurs

Tous les auteurs ont et approuvé la version finale du manuscrit.

## Remerciements

Les auteurs remercient la firme TERUMO BCT pour leur don de matériel et leur appui technique et la fondation Roho Marc Derluyn asbl pour leur appui au programme de chirurgie cardiaque à l’Hôpital Général de Douala.
